# Efficacy and safety of sintilimab combined with trastuzumab and chemotherapy in HER2-positive advanced gastric or gastroesophageal junction cancer

**DOI:** 10.3389/fimmu.2025.1545304

**Published:** 2025-02-14

**Authors:** Zeyu Liu, Aina Liu, Ming Li, Jinyu Xiang, Guohua Yu, Ping Sun

**Affiliations:** ^1^ Department of Oncology, The Affiliated Yantai Yuhuangding Hospital of Qingdao University, Yantai, China; ^2^ Department of Pathology, The Affiliated Yantai Yuhuangding Hospital of Qingdao University, Yantai, China

**Keywords:** HER2-positive, sintilimab, anti-PD-1 antibody, trastuzumab, gastric/gastroesophageal junction cancer, *Helicobacter pylori*

## Abstract

**Background:**

To evaluate the efficacy and safety of sintilimab in combination with trastuzumab and chemotherapy for HER2-positive advanced gastric/gastroesophageal junction cancer (GC/GEJC).

**Methods:**

HER2-positive advanced GC/GEJC patients admitted to our department between January 2018 and October 2024 were included in this study. Patients who received sintilimab in combination with trastuzumab and chemotherapy were assigned to cohort A, while patients who received trastuzumab and chemotherapy alone were assigned to cohort B. The primary endpoints were progression-free survival (PFS) and overall survival (OS), while the secondary endpoints included disease control rate (DCR), objective response rate (ORR), and safety.

**Results:**

A total of 103 patients were analyzed, with 46 in cohort A and 57 in cohort B. The ORR was 65.2% in cohort A compared to 40.4% in cohort B, while the DCR was 87.0% in cohort A and 70.2% in cohort B. The median follow-up duration was 14.0 months. Median PFS (mPFS) was 9.4 months (95% CI: 5.6–13.2) for cohort A and 7.4 months (95% CI: 6.1–8.7) for cohort B (p = 0.089). Median OS (mOS) was 16.4 months (95% CI: 11.5–21.3) in cohort A versus 14.2 months (95% CI: 11.2–17.2) in cohort B (p = 0.069). Adverse events were predominantly mild, and no treatment-related deaths occurred.

**Conclusion:**

Sintilimab combined with trastuzumab and chemotherapy showed promising efficacy and acceptable safety in HER2-positive advanced GC/GEJC. However, no statistically significant improvement in survival outcomes was observed compared to trastuzumab and chemotherapy alone.

## Introduction

Gastric cancer (GC) is the fifth most common cancer and the fifth leading cause of cancer-related mortality worldwide (1). In China, GC ranks as the third leading cause of cancer-related mortality and accounts for 44% of global GC cases, with more than 80% of these cases diagnosed as advanced carcinomas (2–4). In patients with advanced GC, conventional chemotherapy approaches have been reported to yield median overall survival (mOS) ranging from 10 to 12 months (5).

Immunotherapy has emerged as a breakthrough in the treatment landscape of gastric and gastroesophageal junction adenocarcinoma. Based on the KEYNOTE-059 (6) and ATTRACTION-02 (7) trials, nivolumab and pembrolizumab have been successively approved for third-line treatment of recurrent or metastatic gastric/GEJ adenocarcinoma. Results from the CheckMate 649 and ATTRACTION-4 trials demonstrated the efficacy of immunotherapy combined with chemotherapy in the first-line treatment of GC, with prolonged patient survival (8, 9). In GC patients exhibiting HER2 overexpression, observed in 7.3% to 20.2% of cases, standard chemotherapy regimens are typically associated with a poor prognosis (10). Trastuzumab is a monoclonal antibody that targets the HER2 receptor and can interact with natural killer (NK) cells to upregulate PD-L1 expression in GC cells (11). Trastuzumab combined with chemotherapy can significantly improve the survival of HER2-positive advanced GC patients and has been recommended as the first-line treatment for these patients (12–14). In addition, anti-programmed death-1 receptor (PD-1) antibodies can enhance trastuzumab-induced T-cell-specific immune responses, thereby producing synergistic effects when combined with trastuzumab. A phase III trial indicated that the combination of pembrolizumab with trastuzumab and chemotherapy improved progression-free survival (PFS) and overall survival (OS) in GC patients, particularly in those with a PD-L1 combined positive score (CPS) ≥1 (15–17).

Sintilimab is a recombinant humanized anti-PD-1 antibody with higher affinity and a slower dissociation rate for PD-1 compared to pembrolizumab and nivolumab. It demonstrates high PD-1 occupancy both *in vitro* and *in vivo*. The slower dissociation rate allows Sintilimab to maintain binding site occupancy for extended periods even as plasma concentrations decline, potentially leading to prolonged drug activity. Additionally, Sintilimab exhibits a lower risk of immunogenicity relative to pembrolizumab and nivolumab (18). One study demonstrated that sintilimab exhibited a satisfactory efficacy and safety profile in advanced GC/GEJC when combined with chemotherapy (mOS, 15.2 vs. 12.3 months) (19). However, the efficacy and safety of sintilimab combined with trastuzumab and chemotherapy remain unknown. Thus, we conducted a retrospective cohort study to evaluate the efficacy and safety profiles of sintilimab in combination with trastuzumab and chemotherapy for HER2-positive advanced GC patients. Additionally, this study aimed to determine the factors influencing the efficacy for GC patients, such as PD-L1 CPS scores and H. pylori infection, among others (20, 21).

## Methods

### Study design and patients

This is a retrospective cohort study conducted at The Affiliated Yantai Yuhuangding Hospital of Qingdao University. The study was conducted from January 2018 to October 2024. HER2-positive advanced GC/GEJC patients who received either sintilimab in combination with trastuzumab and chemotherapy or trastuzumab and chemotherapy alone were included in this study and were defined as Cohort A and Cohort B, respectively. The inclusion criteria were as follows: age ≥18 years, HER2 immunohistochemistry (IHC) score 3+ or 2+ with fluorescence *in situ* hybridization (FISH)+, microsatellite instability-low/microsatellite stability/proficient mismatch repair, Epstein-Barr virus negative, Eastern Cooperative Oncology Group (ECOG) performance status ≤2, and measurable lesions according to the Response Evaluation Criteria in Solid Tumors (RECIST) version 1.1. The exclusion criteria were as follows: previous receipt of any anti-tumor therapy except for adjuvant or neoadjuvant treatment, and presence of serious or uncontrollable systemic diseases.

### Treatments

Patients received 200 mg of sintilimab intravenously once every three weeks. Trastuzumab was also administered intravenously, starting with a loading dose of 8 mg/kg, followed by a maintenance dose of 6 mg/kg every three weeks. First-line chemotherapy was administered according to the following regimen: patients received capecitabine (1000 mg/m², twice daily for 14 days, followed by a 7-day rest period) or tegafur (40 mg/m², twice daily for 14 days, followed by a 7-day rest period), in combination with oxaliplatin (130 mg/m² on day 1). The chemotherapy cycle was repeated every three weeks. After six to eight cycles of treatment, patients underwent maintenance therapy with sintilimab combined with trastuzumab and capecitabine or tegafur until disease progression.

### Efficacy and safety assessments

Efficacy was assessed every two cycles based on RECIST 1.1. Response to treatment was categorized as complete response (CR), partial response (PR), stable disease (SD), or progressive disease (PD). The primary endpoints were PFS and OS. PFS was defined as the time from initial treatment to disease progression or death, while OS was defined as the time from initial treatment to death from any cause. Secondary endpoints included the objective response rate (ORR), disease control rate (DCR), and safety. ORR was defined as the proportion of patients achieving a PR or CR as their best response. DCR was defined as the proportion of patients achieving a PR, CR, or SD as their best response. Adverse events (AEs) were evaluated according to the Common Terminology Criteria for Adverse Events (CTCAE), version 4.0.

### Statistical analysis

All data were analyzed using SPSS version 27.0 (IBM, New York, USA). Categorical variables were compared using χ² tests and Fisher’s exact tests. PFS and OS were calculated using the Kaplan-Meier method, and results were presented with the median and 95% confidence intervals (CIs). Univariate Cox regression was used to calculate hazard ratios (HR). To identify factors affecting efficacy, we created several subgroups based on PD-L1 CPS scores, ECOG status, and H. pylori infection, among others. Survival analyses between cohorts or subgroups were performed using the log-rank test. All tests were two-sided, with a significance level of p<0.05.

## Result

### Patient characteristics

**Figure 1:**
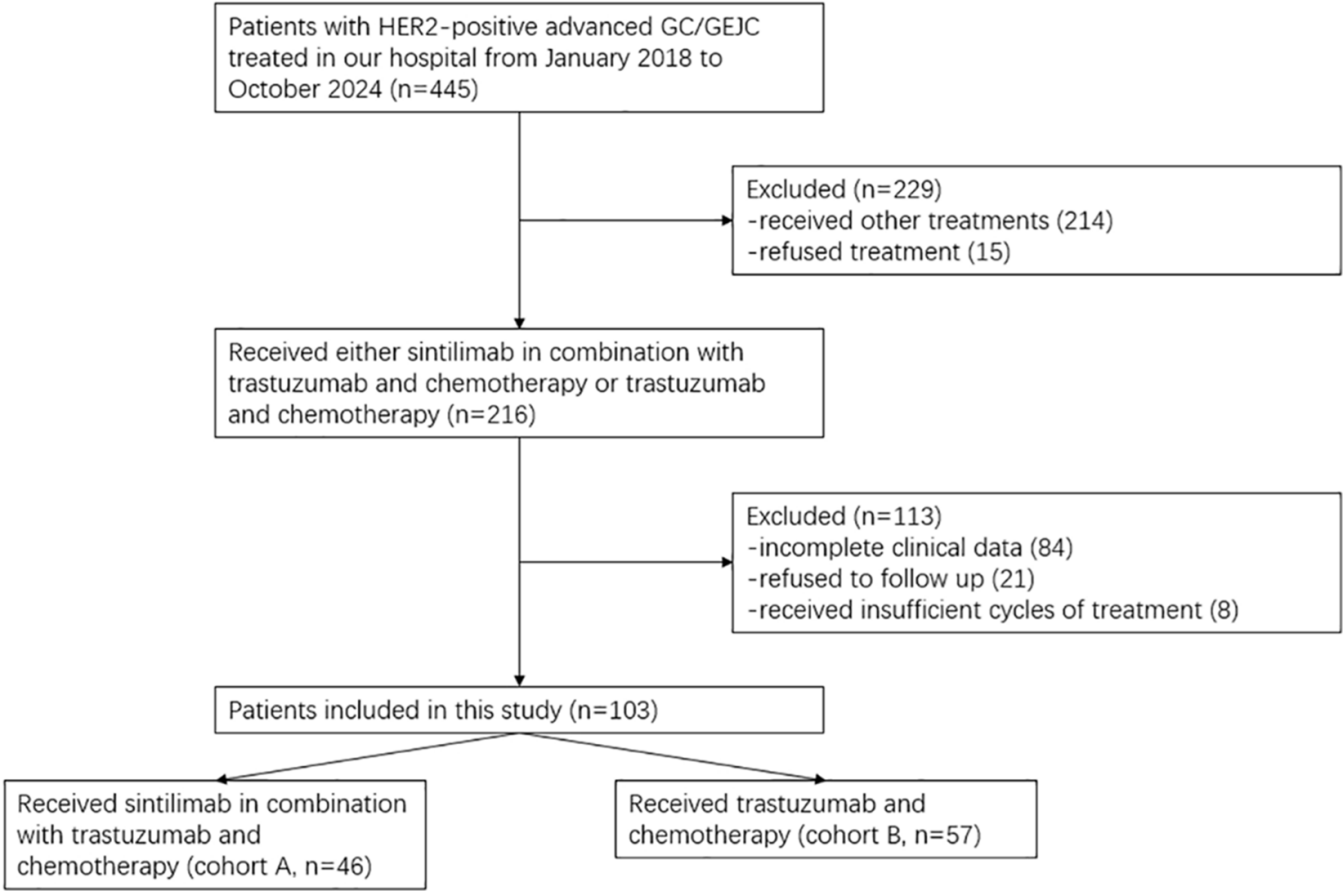
Flowchart of the recruitment process.

A total of 103 patients were enrolled in this study from January 2018 to October 2024. Of these, 46 patients received sintilimab in combination with trastuzumab and chemotherapy and were assigned to cohort A, while 57 patients who received trastuzumab and chemotherapy alone were assigned to cohort B. The recruitment process of our study is shown in **Figure 1**. The median follow-up durations for cohort A and cohort B were 14.3 months and 13.0 months, respectively. The patients in cohort A and cohort B were comparable in terms of age distribution, ECOG performance status, sex distribution, and other factors (**Table 1**). A total of 15 patients discontinued therapy due to adverse events (AEs), poor health, or personal reasons. The median treatment cycle was 6 cycles.

**Table 1:** Clinical characteristics of the study population.

Clinical characteristic	Cohort A (n=46)	Cohort B (n=57)	P value
Age (years; n, %)			0.616
<60	20 (43.5)	22 (38.6)	
≥60	26 (56.5)	35 (61.4)	
ECOG performance status (n, %)			0.752
0-1	42 (91.3)	53 (93.0)	
2	4 (8.7)	4 (7.0)	
Sex (n, %)			0.122
Female	15 (32.6)	11 (19.3)	
Male	31 (67.4)	46 (80.7)	
Liver metastasis (n, %)			0.070
No	26 (56.5)	22 (38.6)	
Yes	20 (43.5)	35 (61.4)	
Peritoneal metastasis (n, %)			0.786
No	43 (93.5)	54 (94.7)	
Yes	3 (6.5)	3 (5.3)	
Primary tumor location (n, %)			0.671
GEJ	3 (6.5)	5 (8.8)	
Gastric	43 (93.5)	52 (91.2)	
H. pylori (n, %)			0.464
Negative	30 (65.2)	41 (71.9)	
Positive	16 (34.8)	16 (28.1)	
PD-L1 CPS scores (n, %)			0.378
<1	21 (45.7)	31 (54.4)	
≥1	25 (54.3)	26 (45.6)	
HER2 status (n, %)			0.276
IHC (2+) and FISH (+)	14 (30.4)	12 (21.1)	
IHC (3+)	32 (69.6)	45 (78.9)	
Hemoglobin (n, %)			0.094
Low	27 (60.9)	24 (42.1)	
Normal	19 (39.1)	33 (57.9)	
Albumin (n, %)			0.495
Low	32 (69.6)	36 (63.2)	
Normal	14 (30.4)	21 (36.8)	
Nutritional status* (n, %)			0.420
poor	37 (80.4)	42 (73.7)	
Normal	9 (19.6)	15 (26.3)	

* normal nutritional status were defined as albumin ≥ 40 g/L and hemoglobin ≥120 g/L for males and ≥110 g/L for females prior to treatment.

### Efficacy

**Figure 2:**
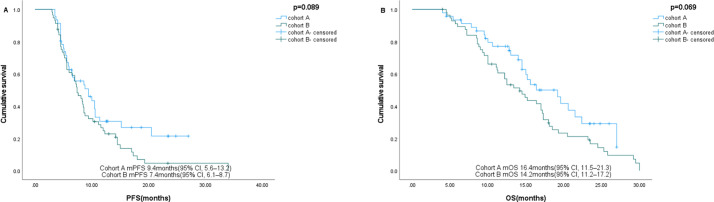
**(A)** PFS for cohort A and cohort B **(B)** OS for cohort A and cohort B.

**Figure 3:**
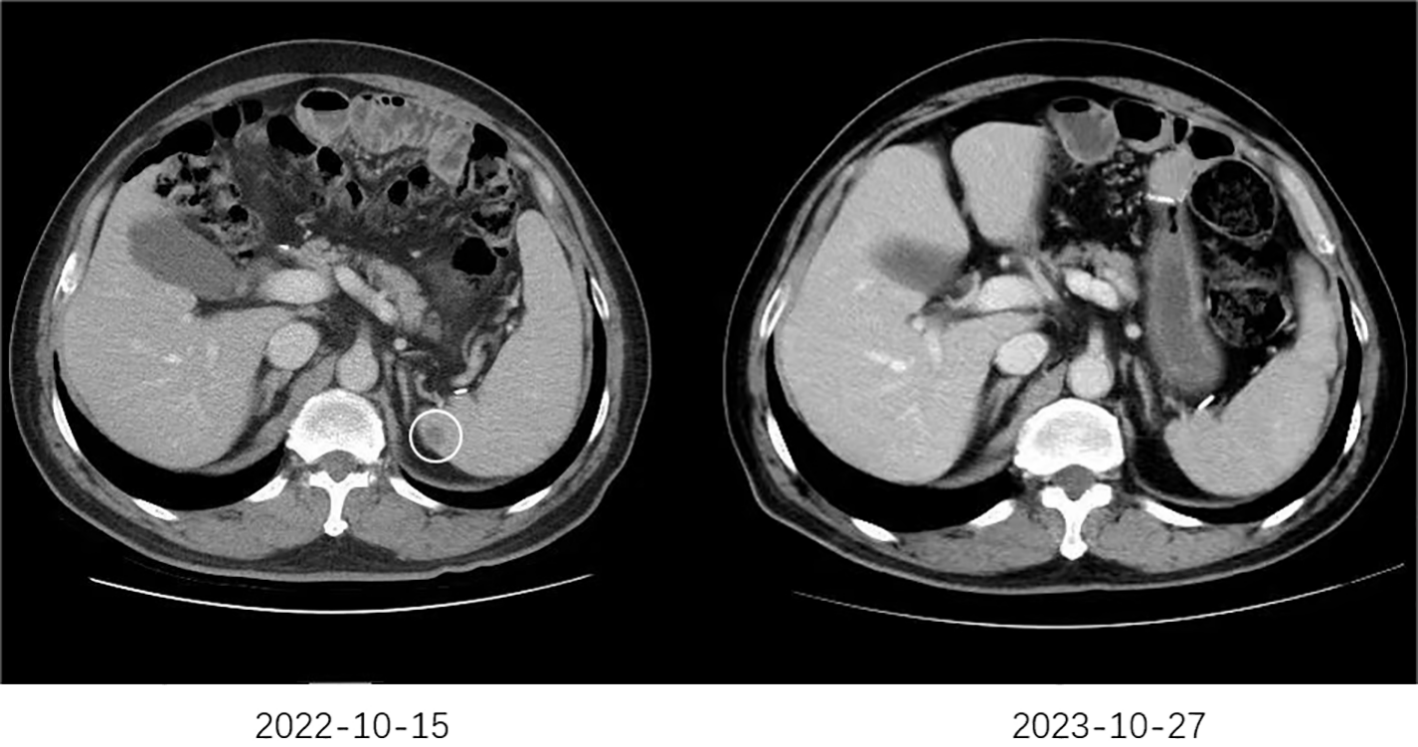
The typical CT images of a CR patient. The CT scan performed on October 15, 2022, revealed newly developed splenic metastases. Subsequently, the patient began first-line treatment on October 18, 2022, consisting of six cycles of sintilimab combined with trastuzumab and chemotherapy. Maintenance therapy with sintilimab, trastuzumab, and chemotherapy was continued thereafter. A follow-up CT scan on October 27, 2023, demonstrated the disappearance of the splenic metastases.

The follow-up cutoff date was 31 October 2024. The median progression-free survival (mPFS) for cohort A and cohort B were 9.4 months (95% CI, 5.6–13.2 months) and 7.4 months (95% CI, 6.1–8.7 months), respectively (**Figure 2A**). The mOS for cohort A and cohort B were 16.4 months (95% CI, 11.5–21.3 months) and 14.2 months (95% CI, 11.2–17.2 months), respectively (**Figure 2B**). Among the 46 patients in cohort A, one (2.2%) achieved a complete response (CR), 29 (63.0%) achieved a partial response (PR), 10 (21.7%) had stable disease (SD), and six (13.0%) had progressive disease (PD). Of the 57 patients in cohort B, 23 (40.4%) achieved PR, 17 (29.8%) had SD, and 17 (29.8%) had PD. Cohort A showed a higher overall response rate (ORR) of 65.2% compared to 40.4% in cohort B (P=0.012), and a higher disease control rate (DCR) of 87.0% compared to 70.2% in cohort B (P=0.042) (**Table 2**). **Figure 3** shows typical and clear computed tomography (CT) images of a CR patient from Cohort A before and after treatment.

**Table 2:** Treatment response based on RECIST 1.1.

Efficacy	Cohort A (n=46)	Cohort B (n=57)	P value
CR (n, %)	1 (2.2)	0 (0)	–
PR (n, %)	29 (63.0)	23 (40.4)	–
SD (n, %)	10 (21.7)	17 (29.8)	–
PD (n, %)	6 (13.0)	17 (29.8)	–
ORR (%)	65.2	40.4	0.012
DCR (%)	87.0	70.2	0.042

### Subgroup analysis

**Figure 4:**
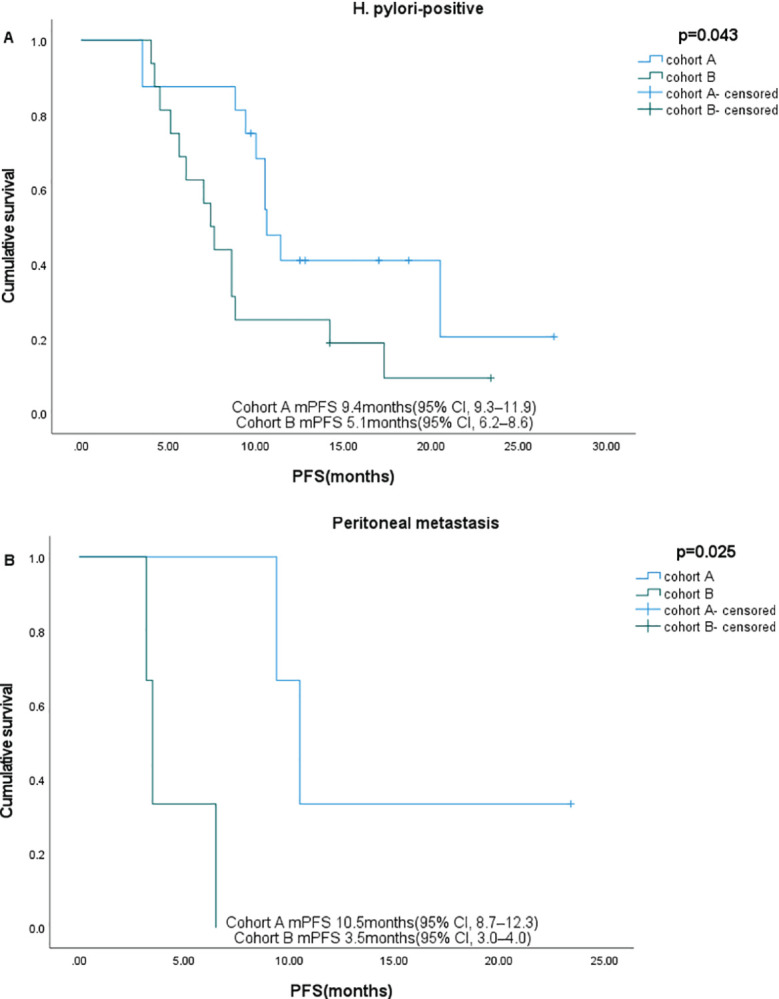
Subgroup analysis of PFS in different subgroups. **(A)** H pylori-positive, **(B)** Peritoneal metastases.

**Figure 5:**
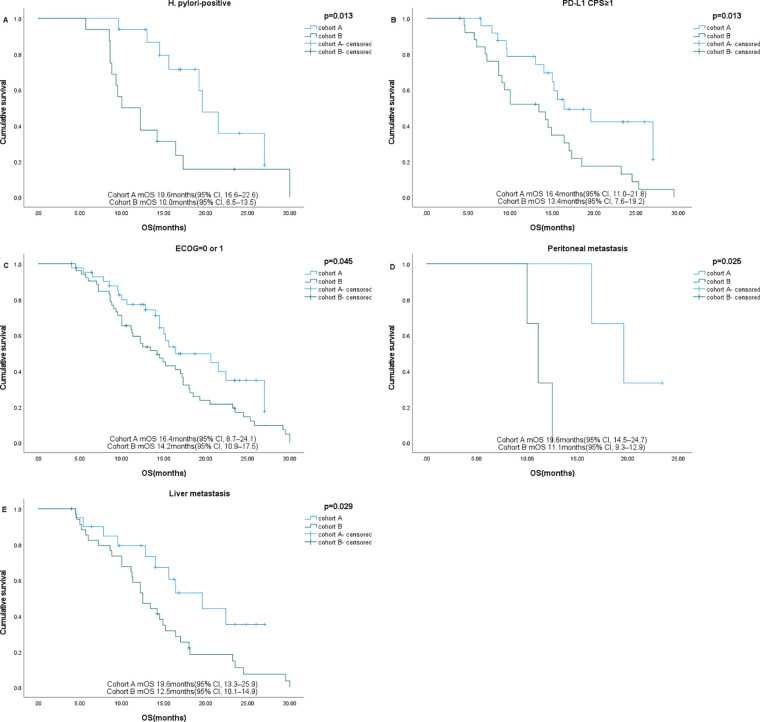
Subgroup analysis of OS in different subgroups. **(A)** H pylori-positive, **(B)** PD-L1 CPS ≥1, **(C)** ECOG=0-1, **(D)** Peritoneal metastases **(E)** Liver metastases.

Subgroup analysis showed that gender, age, HER2 status, and nutritional status were not associated with patient survival. Among patients with ECOG ≤1, liver metastases, and PD-L1 CPS ≥1, those who received sintilimab had better OS. In patients with peritoneal metastases and H. pylori-positive status, sintilimab provided significant benefits in both PFS and OS (**Figures 4**, **5**, **Table 3**).

**Table 3:** Summary of Median Progression-Free Survival (mPFS), Median Overall Survival (mOS), and Hazard Ratios (HR) for Cohorts A and B.

Group	Median (months)	95% CI (months)	P value	HR (95% CI)
Overall (PFS)			0.089	1.460 (0.937-2.276)
Cohort A	9.4	5.6-13.2		
Cohort B	7.4	6.1-8.7		
Overall (OS)			0.069	1.560 (0.959-2.537)
Cohort A	16.4	11.5-21.3		
Cohort B	14.2	11.2-17.2		
H. pylori-positive (PFS)			0.043	2.283 (0.998-5.223)
Cohort A	9.4	9.3-11.9		
Cohort B	5.1	6.2-8.6		
H. pylori-positive (OS)			0.013	2.933 (1.203-7.149)
Cohort A	19.6	16.6-22.6		
Cohort B	10.0	6.5-13.5		
Peritoneal metastasis (PFS)			0.025	–
Cohort A	10.5	8.7-12.3		
Cohort B	3.5	3.0-4.0		
Peritoneal metastasis (OS)			0.025	–
Cohort A	19.6	14.5-24.7		
Cohort B	11.1	9.3-12.9		
PD-L1 CPS≥1 (OS)			0.013	2.305 (1.165-4.561)
Cohort A	16.4	11.0-21.8		
Cohort B	13.4	7.6-19.2		
ECOG=0 or 1 (OS)			0.045	1.689 (1.003-2.844)
Cohort A	16.4	8.7-24.1		
Cohort B	14.2	10.9-17.5		
Liver metastasis (OS)			0.029	2.177 (1.061-4.465)
Cohort A	19.6	13.3-25.9		
Cohort B	12.5	10.1-14.9		

### Safety

In cohort A, the most common treatment-related adverse events (AEs) were bone marrow suppression (18/46, 39.1%) and abnormal liver function (12/46, 26.1%). The most common grade 3-4 AE was bone marrow suppression (6/46, 13.0%). In cohort B, the most common treatment-related AEs were bone marrow suppression (19/57, 33.3%), abnormal liver function (10/57, 17.5%), and diarrhea (10/57, 17.5%). The most common grade 3-4 AE was bone marrow suppression (4/57, 7.0%). No treatment-related deaths were observed, and there was no statistically significant difference in the incidence of treatment-related AEs between the two cohorts. AEs that occurred during treatment in cohort A and B were summarized in **Table 4**.

**Table 4:** Treatment-related adverse events (AEs) occurred during treatments.

Treatment-related AEs	Cohort A	Cohort B		P value
Bone marrow suppression	18 (39.1)	19 (33.3)		0.542
Grade < 3	12 (26.1)	15 (26.3)		
Grade ≥ 3	6 (13.0)	4 (7.0)		
Abnormal liver function	12 (26.1)	10 (17.5)		0.293
Grade < 3	11 (23.9)	9 (15.8)		
Grade ≥ 3	1 (2.2)	1 (1.8)		
Diarrhea	10 (21.7)	10 (17.5)		0.593
Grade < 3	10 (21.7)	10 (17.5)		
Grade ≥ 3	0 (0)	0 (0)		
Rash	10 (21.7)	7 (12.3)		0.199
Grade < 3	10 (21.7)	7 (12.3)		
Grade ≥ 3	0 (0)	0 (0)		
Pyrexia	10 (21.7)	7 (12.3)		0.199
Grade < 3	8 (17.4)	6 (10.5)		
Grade ≥ 3	2 (4.3)	1 (1.8)		
Nausea and vomiting	9 (19.6)	7 (12.3)		0.310
Grade < 3	8 (17.4)	6 (10.5)		
Grade ≥ 3	1 (2.2)	1 (1.8)		
Hand-foot syndrome	9 (19.6)	6 (10.5)		0.196
Grade < 3	7 (15.2)	5 (8.8)		
Grade ≥ 3	2 (4.3)	1 (1.8)		
Hypokalemia	8 (17.4)	5 (8.8)		0.190
Grade < 3	7 (15.2)	5 (8.8)		
Grade ≥ 3	1 (2.2)	0 (0)		
Decreased appetite	6 (13.0)	4 (7.0)		0.336
Grade < 3	6 (13.0)	4 (7.0)		
Grade ≥ 3	0 (0)	0 (0)		
Neurotoxicity	6 (13.0)	4 (7.0)		0.336
Grade < 3	5 (10.9)	3 (5.3)		
Grade ≥ 3	1 (2.2)	1 (1.8)		
Fatigue	5 (10.9)	4 (7.0)		0.728
Grade < 3	5 (10.9)	4 (7.0)		
Grade ≥ 3	0 (0)	0 (0)		
Stomatitis	4 (8.7)	3 (5.3)		0.728
Grade < 3	4 (8.7)	3 (5.3)		
Grade ≥ 3	0 (0)	0 (0)		
Hypothyroidism	2 (4.3)	0 (0)		0.197
Grade < 3	2 (4.3)	0 (0)		
Grade ≥ 3	0 (0)	0 (0)		
Pneumonitis	1 (2.2)	1 (1.8)		1.000
Grade < 3	1 (2.2)	1 (1.8)		
Grade ≥ 3	0 (0)	0 (0)		

## Discussion

Camrelizumab and pembrolizumab, have been shown to generate longer survival periods for HER2-positive advanced GC patients when combined with trastuzumab and chemotherapy (22, 23). This is, to our knowledge, the first study to explore the efficacy of sintilimab in combination with trastuzumab and chemotherapy for the treatment of HER2-positive advanced GC patients.

At the European Society for Medical Oncology (ESMO) annual meeting in September 2024, the KEYNOTE-811 trial presented final data. The mOS was prolonged by 3.2 months in the pembrolizumab group compared to the control group (20.0 months vs. 16.8 months, HR=0.80, 95% CI 0.67–0.94, P=0.004). In patients with PD-L1 CPS ≥1, the mOS was 20.1 months in the pembrolizumab group and 15.7 months in the control group (HR=0.79, 95% CI 0.66–0.95, P=0.006) (15). Our study yielded similar results and further confirmed the satisfactory efficacy of anti-PD-1/PD-L1 therapy combined with anti-HER2 therapy and chemotherapy in patients with PD-L1 CPS ≥1. However, the comparison of overall survival (OS) results between the two cohorts in our study differed from those of the KEYNOTE-811 trial, possibly due to the differing proportions of patients with PD-L1 CPS ≥1 in the two studies (49.5% vs. 84.7%).

In subgroup analysis, sintilimab prolonged PFS in patients with peritoneal metastases or H. pylori positivity and prolonged OS in patients with ECOG ≤1, peritoneal metastases, liver metastases, H. pylori positivity, or PD-L1 CPS ≥1. 15 patients (32.6%) in Cohort A and 20 patients (35.1%) in Cohort B received subsequent therapy, including new chemotherapy, targeted therapies, or immunologic agents. These patients had better survival outcomes than those who did not receive subsequent therapy. DS8201 has been approved for the treatment of advanced GC, providing a promising therapeutic option for this patient population. Based on current evidence-based medical guidelines and drug availability, we consider DS8201 as a third-line and subsequent treatment for HER2-positive advanced GC patients who have previously experienced failure with trastuzumab therapy. The efficacy of DS8201 in this setting will be further evaluated through ongoing data collection and analysis in future studies.

Patients with PD-L1 CPS ≥1 demonstrated better therapeutic efficacy. Trastuzumab increased HER2 internalization and enhanced cross-presentation in dendritic cells, a process specifically mediated by its Fc receptor interaction. This mechanism subsequently increased antigen-specific antigen-specific cytotoxic T lymphocytes generation both *in vitro* and *in vivo* through improved cross-presentation by dendritic cells (24). Second, CD4+ T cells must recognize peptide-histocompatibility complex class II (MHC-II) complexes to become activated. Trastuzumab upregulates major MHC-II molecules in the tumor microenvironment, increasing antigen availability for CD4+ T-cell activation (11, 25). In particular, it enhances the expression of PD-1/PD-L1, thereby potentiating the efficacy of anti-PD-1 antibodies (25). Specifically, trastuzumab upregulates PD-L1 levels in HER2-overexpressing cancer cells by activating the extrinsic pathway, where immune effector cells release IFNγ. Moreover, IFN-γ can help create an environment that conducive to tumor-specific adaptive immunity. These effects are amplified in patients with higher PD-L1 CPS, as trastuzumab enhances PD-1/PD-L1 expression, further synergizing with anti-PD-1 antibodies like sintilimab (26, 27). Through these mechanisms, sintilimab and trastuzumab promote the expansion of peripheral memory T-cells, synergistically prolonging the OS of patients (28).

Our study is the first to demonstrate that among HER2-positive patients with advanced GC, those who are H. pylori-positive achieve improved survival outcomes following combined treatment with sintilimab, trastuzumab, and chemotherapy. H. pylori CagA can elevate PD-L1 expression in exosomes derived from GC cells, resulting in overall higher PD-L1 levels in H. pylori-positive versus H. pylori-negative patients (29, 30). In line with these findings, Jia et al. observed that H. pylori–positive GC may exhibit elevated densities of PD-L1+ and PD-1+ T cells, increased PDCD1 and CD274 gene expression, and a tumor microenvironment enriched with more nonexhausted CD8+ T cells. Moreover, gene set variation analysis (GSVA) indicates that H. pylori-positive tumors have a heightened proliferation-related score and a reduced stroma-related score, both of which have been linked to enhanced responses to immunotherapy (31). These findings collectively suggest that H. pylori infection fosters a tumor microenvironment conducive to immunotherapeutic efficacy, and potential biomarkers such as PD-1/PD-L1 (PDCD1/CD274) expression levels and proliferation/stroma-associated signatures may help guide treatment strategies in this patient population. Moreover, Their study demonstrates that H. pylori infection enhances the therapeutic benefit of immunotherapy in patients with PD-L1 CPS <1 or <5. Notably, the advantage of immunotherapy persists in H. pylori-positive GC patients, even in the absence of PD-L1 expression (32). Jia et al. therefore concluded that H. pylori infection is a favorable prognostic indicator for immunotherapy in GC patients, a conclusion that is consistent with our own results.

Patients with liver and peritoneal metastases had better survival after receiving the combined therapy, which is consistent with other studies (33). Subgroup analyses in the CheckMate-649 and KEYNOTE-859 trials showed statistically significant improvements in median OS in patients with liver metastases (34, 35). However, the molecular mechanisms remain to be further elucidated.

However, several limitations must be acknowledged. First, as a retrospective analysis, the study was hindered by inherent challenges, including incomplete patient treatment records that restricted the breadth of available data. Beyond the clinical factors included in our study, other variables such as Tumor Mutation Burden (TMB) may influence patient survival outcomes. However, due to economic constraints and other factors, we were unable to obtain sufficient TMB data for comprehensive analysis in our retrospective cohort. Second, the relatively small sample sizes in both cohorts limit the generalizability of our results and may introduce potential biases in subgroup analyses. Furthermore, the specific design of our subgroup analyses, which involved comparing survival outcomes within distinct patient subsets (e.g., H. pylori-positive patients) between Cohort A and Cohort B, precluded the feasibility of performing multivariate Cox regression analyses, as such analyses would require overlapping or larger populations that are not available in our current sample. This constraint underscores the necessity for larger, more diverse patient populations and more comprehensive data collection in future research. We are currently planning a prospective, multicenter trial that will not only validate our findings but also clarify the underlying mechanisms by which clinical factors influence immunotherapy responses. Finally, the current investigation did not explore the underlying mechanisms of how clinical factors influence immunotherapy responses, representing a critical avenue for future inquiry.

In conclusion, there were no statistically significant differences between sintilimab in combination with trastuzumab and chemotherapy and trastuzumab with chemotherapy for overall HER2-positive advanced GC/GEJC patients. However, sintilimab in combination with trastuzumab and chemotherapy may result in better OS in patients with PD-L1 CPS ≥1, liver metastases, or ECOG ≤1. Additionally, in patients with peritoneal metastases and H. pylori-positive status, sintilimab led to better PFS and OS.

## Data Availability

The original contributions presented in the study are included in the article/supplementary material. Further inquiries can be directed to the corresponding authors.
